# Intersectionality, special populations, needs and suggestions: the Flint Women’s study

**DOI:** 10.1186/s12939-020-1133-9

**Published:** 2020-01-31

**Authors:** Maji Hailemariam, Julia W. Felton, Kent Key, DeOnica Greer, Bernadel L. Jefferson, Janice Muhammad, Raven Miller, Fallon Richie, DeWaun Robinson, Sharon Saddler, Bryan Spencer, Monicia Summers, Jonne Mc Coy White, Jennifer E. Johnson

**Affiliations:** 1 Division of Public Health, College of Human Medicine, Michigan State University, Flint, MI USA; 2 Community Based Organization Partners, Flint, MI USA; 3 Community resident, Flint, MI USA; 4 Combined-Integrated Clinical and Counseling Program, University of South Alabama, Mobile, AL USA; 5 My Exceptionality LLC, Flint, MI USA

## Abstract

**Background:**

Equitable access to services that promote health and wellbeing is an important component of social justice. A community-engaged participatory qualitative study was conducted in Flint, Michigan, USA, to understand the needs of special populations (young women, perinatal women and new mothers, older women, women with disabilities, and LGBTQIA women) and elicit their ideas about solutions.

**Methods:**

In-depth interviews (*n* = 100) were conducted. Participants were either women living in the Flint area, human service providers in the area, or both. A team of community and academic coders analyzed the data using an a priori framework.

**Results:**

Participants identified needs of different groups of women and suggested ways to address them. Access to healthy food, reducing healthcare costs, and improving transportation, job opportunities and affordable quality housing were crosscutting themes across all groups of women. Mentoring support was said to protect vulnerable young women from the risk of human trafficking. Older women were said to gain a sense of purpose, build their social support and reduce their loneliness by engaging in mentoring younger women. Women with disabilities were reported to benefit from infrastructure accessibility and authentic inclusion in all areas of life. Providing help that considers their dignity, pride and self-worth were suggested. LGBTQIA women were reported to have housing needs due to discrimination; mostly turned down as renters and can be rejected from faith-based homeless shelters. LGBTQIA women would also benefit from increased sensitivity among healthcare providers. For all groups of women, streamlining access to social services and other resources, building social support networks and increasing awareness about existing resources were recommended.

**Conclusion:**

Efforts directed towards improving women’s health and wellbeing should include perspectives and suggestions of diverse groups of women from the community. Acting on suggestions that emanate from the community’s lived experiences may reduce inequalities in health and wellbeing.

## Background

Equitable access to services that promote health and wellbeing is an important component of social justice. Social justice in the context of access to health care and other services includes equal access for equal need and equal utilization of services leading to improved outcomes once enrolled [1]. Individuals’ experiences of health and wellbeing are often shaped and framed by their place in the society. Vulnerable groups are defined as specific sections of the society who have limited access to resources and disproportionately experience premature mortality and morbidity [2]. Discrimination based on socioeconomic status, gender, disability, age and sexual orientation can have a significant negative impact on vulnerable populations in relation to access to services. Poor access to resources and exposure to risks contribute to poor health and overall wellbeing [3]. In response to this, population-based approaches have been widely advocated to create equity of access.

Flint, Michigan, U.S.A. faces social, health and economic challenges. The city struggles with high poverty rates, unemployment and lack of economic opportunities. In recent years, Flint has suffered significant depopulation and subsequent decreases in revenue and increases in abandoned houses and blight [4, 5]. After the Water Crisis that disproportionately affected racial and ethnic minorities [6], Flint has been mentioned as a classic case of environmental injustice [7–9]. However, Flint also represents a strong and thriving community that is flourishing in the face of various challenges. Flint has a long positive history of community activism, beginning with the sit-down strike of 1937, which led to the unionization of the US automobile industry. This community activism and involvement has strengthened over time in response to health and other needs [10]. For example, there is an active community body reviewing the ethical conduct of research involving Flint residents [11, 12]. The community is also highly engaged in responding to the Flint water crisis [6, 10] and leading research efforts directed towards improving health outcomes for its residents [13, 14].

The Flint Women’s Study builds on the existing history of community activism and involvement. The Flint Women’s Study [15] was a large (*n* = 100) community-engaged qualitative research conducted to identify the needs, hopes, dreams and strengths of Flint area women in order to design community-partnered approaches to address them better. In particular, the study sought the suggestions of Flint-area women about how to mobilize individual and community strengths to better address their needs.

Intersectionality theory describes how multiple, interconnected and interlocking systems of power affect individuals who are less privileged in the society [16]. Forms of social stratification may include class, race, sexual orientation, age, religion, disability, and gender, and are often interwoven. For example, most respondents in the Flint Women’s Study were female, a majority belonged to racial and ethnic minority groups, many were poor, and some also belonged to or represented other marginalized or historically disempowered groups. In recent years, intersectionality theory has been increasingly used as a framework to explain how multiple forms of social stratification coexist and affect individuals and communities. The concept has been used to explain a range of issues including social identities, power structures, and legal and policy frameworks [17]. In the present study, we used intersectionality theory as an a priori framework to analyze the unmet needs experienced by specific groups of women (young women, perinatal women and new mothers, older women, women with disabilities and LGBTQIA).

The purpose of this study was to examine needs of special populations and propose ways to address these unmet needs. We present the recommendations from women in the Flint community about how to improve the health and wellbeing of some of its vulnerable members. Disparities in access to services were examined by life stage, sexual orientation and gender identities, and disability status.

## Methods

### Rationale for the Flint Women’s study

Flint, Michigan is one of the Rust Belt cities that experienced outmigration, decline in economic activities, violent crime and various health needs [4, 18, 19]. Women and girls are disproportionately affected by these challenges. The Flint Women’s Study originated from informal discussions the last author (JJ) held with Flint community partners with whom she has previous research collaborations. The original plan was to have a conversation with women from different walks of life to understand their hopes, needs, strengths and challenges and use the information to design ways to improve their health and wellbeing. The community partners suggested focusing on the different life-stage challenges, disability, gender identity and sexual orientation. The community partners also contributed to the study design, topic guide development, participant recruitment and data analysis. Details of the partnership process and the rationale is published elsewhere [20].

### Study design

The current analysis focuses on women’s perspectives on how to improve the health and wellbeing of subpopulations of women in the Flint area (young women, perinatal women and new mothers, older women, women with disabilities, and LGBTQIA women). The study follows a thematic analysis whereby the process of routine data collection and analysis are not mutually exclusive. Thematic analysis provides a flexible and pragmatic approach whereby researchers can draw patterns and relationships within and between themes and revise and add themes as they emerge throughout the analysis [21].

### Sampling

A combination of purposive and snowball sampling was used. Inclusion criteria were: (1) age 18 or older, and (2) be a woman living in the Flint area (i.e., Genesee County, the county that houses Flint) and/or someone who serves women living in the Flint area. We posted flyers inviting people to participate in the study in public places such as residence areas, social service offices and churches. We reached out to participants through the networks of our community partners. We also contacted individuals leading and receiving services in social service agencies and women we met on the streets. At the end of each interview, participants were asked to suggest another person whose perspectives they think might be relevant to fulfil the study objectives and enrich the data. The goal was to include as broad a range of participants as possible. Halfway through the 100 interviews, we checked backgrounds of our interviewees to reduce the risk of selection bias and ensure that our participants represented a broad and diverse range of women. We modified our selection criteria to identify individuals who were underrepresented in our study (eg. Muslim women, women with disabilities, LGBTQIA women, sex workers etc.). Consenting individuals were invited to participate in a face-to-face interview.

### Data collection

Rationale for the design and methods of the Flint Women’s Study [15] is published elsewhere [20]. Open-ended topic guides were developed by one of the authors (JJ) in partnership with the other authors, which included both academic and community contributors. The topic guides explored needs and solutions for women in general, as well as needs and solutions for several special populations of women, including young women, perinatal women and new mothers, older women, women with disabilities, and LGBTQIA women. The topic guides were generic. However, specific probes were added to tailor the interviews to the interest or expertise of the interviewees. Participants were asked to suggest questions they think we should’ve raised. They suggested including questions about the needs of women with disabilities and LGBTQIA women. These questions were incorporated to the interview guide at an earlier stage of the data collection. Questions also covered service gaps, existing resources and what is required to address these unmet needs. Interviews were collected by two of the authors (JJ and MH) from August 2017–February 2018. Both interviewers have extensive qualitative experience including designing and leading large qualitative studies. JJ led interviewing of 75% of the participants. MH conducted the remaining 25 interviews independently. After each interview, the interviewers summarized their notes in a spreadsheet detailing key aspects of each interview. Interviews were conducted face-to-face in different locations preferred by the study participants, including homes of participants, homeless shelters, workplaces of participants and offices of researchers (JJ & MH). Privacy of the interview location was considered in all cases. Interviews lasted from 60 to 90 min. All interviews were audio recorded.

### Data analysis

All audio files were transcribed verbatim. Interview transcripts were assigned codes. Names and other personal identifiers were removed from all the transcripts to ensure anonymity. A spreadsheet linking the transcripts with their sociodemographic data was extracted and stored separately. We trained and engaged five pairs of community and academic partners to code the transcripts. The half-day didactic training involved 1) introduction to qualitative coding 2) familiarization with the qualitative coding software 3) orientation to the coding framework and 4) double-coding and addressing disagreements in the coding process. We used NVivo qualitative analysis software, an ideal tool for team-based coding of large scale qualitative data [22]. MH and JJ developed the coding framework. Coders were given an NVivo coding framework to chart data into the broad categories. The coding framework was expanded to incorporate newly emerging themes. The community and academic pairs coded the transcripts assigned to them independently on their separate NVivo files. The pairs held meetings to double-code on a third NVivo file to ensure consensus. All of the transcripts were double-coded. The double-coded NVivo files were merged into the study Masterfile by the first author. We extracted codes under each special population group for analysis to identify the recurring themes. MH identified the recurring themes. JJ and MH discussed and approved the final identified themes for this manuscript.

## Results

A total of 200 people were contacted to participate in the study; 100 were interviewed. Six people refused. Others were not reachable (*n* = 27), undecided (*n* = 28), wrong number or email (*n* = 25) or agreed to participate but did not show up for the interview (*n* = 14). Most (*n* = 94) of the respondents were women. Majority (*n* = 87) of the participants were both women living in the county and who also served women living in the county. Racial composition of the participants was African-American (*n* = 52), white (*n* = 41) and other (*n* = 7), similar to the general population of Flint. Age of participants ranged from 18 to 84 years including young women (*n* = 19), older women (*n* = 15), middle-age women (*n* = 70), young men (*n* = 2), middle age men (*n* = 4). Six women had disabilities. Study participants represented various roles and occupations including counselors, pastors, educators, volunteers, family members, women living in shelters, women in workforce development, librarians, attorneys, judges, students, business leaders, pet groomers and more.

The original interview guide inquired about needs of women across the lifespan. Thus, the study focused on women at different points in their life including 1) young women, 2) perinatal women and mothers 3) older women 4) women with disabilities and 5) LGBTQIA women. Needs and suggestions pertaining to individual, interpersonal and systems/structures issues are presented.

### Young women

The needs of young women and the suggestions to address them fell into two main categories; a) mentoring and positive examples, and b) addressing risky behaviors including sexual behaviors and exposure to risk including human trafficking. Respondents shared concerns about younger women’s body image, low self-esteem, and self- acceptance. Moreover, lack of mentoring and not having role models in the community were said to lead to unhealthy choices and decisions that ultimately have negative impact on young women’s health and wellbeing.

***Mentoring*** was the most frequently endorsed need for young women in the community. Almost all of the young women and participants who worked with young women endorsed the need for mentorship support. One of the participants shared her view as:

*… someone that can guide them or steer them in the right direction. That would be more of like a mentor; and then counseling for all different types of peer pressure issues, ‘cuz there’s so much out here. You got pregnancy. You got drugs. You got abuse, domestic abuse. You got young ladies who even feel as though, maybe, that there’s so much pressure, that they feel as though they don’t have a reason to live, like, suicide will help.* **Entrepreneur, 48**

Another participant expressed her view as:

*… a lot of kids grew up in the foster-care system then, when they’re discharged between 18 and 21 years old, and they’re kind of on their own. If they had someone to kind of tell them what to do, an older mentor, I think that would help.* **Job skills trainer, 33**

Participants highlighted the importance of having supportive, successful examples.

*They need examples. They need individuals that they could look up to as mentors, as a support base, as encouragers, comforters, and supporters. When we look at what’s goin’ on just here in Genesee County, a lot of these young women don't have mothers that are mothers because they’re so busy fightin’ to try to figure out where they fit in as—because they probably started havin’ children at a young age or somethin’ and it just has overtaken them. It’s overwhelmed them, but I think most importantly the young women today need examples*. **Human resources manager, 62**

#### Suggestions for reducing risky behavior and exposure to risk

Participants said that young women want to be loved, approved of, and appreciated. However, they remarked that the need for affection and validation can sometimes lead young women to engage in unhealthy activities or early pregnancy.

*I think so many of them, they just see babies. They’re so cute. They’re so cuddly. I want one. Not realizing no, you really don’t, cuz when they get here, they are here forever. Some girls, they want someone to love them, and they believe this baby will love them unconditionally, or they get caught up in that mentality of if you love me you would. … . They need to learn that instead of playing house, you need to take care of yourself first. Stop playing the role of a wife when you’re just a girl*. **Teacher, 51**

Many participants reported higher rates of teenage pregnancy.

*My cousin has had two babies since we graduated. We graduated the same year. I was in her wedding. We are not even close. I think that says volumes*. **College student, 21**

Ensuring access to contraceptives and addressing misconceptions around different forms of contraceptive options was recommended as a mechanism to reduce teen pregnancy.

*When it comes to birth control, they’re not consciously thinking about it. They’re definitely not consciously thinking about it. That’s part of adolescence, you know? Adolescents have this wonderful vulnerability about them like, “Oh my God. Yeah, I’m gonna have sex, but I’m going get pregnant*.” **Adolescent medical specialist, 58**

Moreover, participants also mentioned that young women held different beliefs and misconceptions regarding contraceptive use. For example, some of the participants stated that young women believe contraceptives cause weight gain or may result in future infertility.

Many participants emphasized the need for comprehensive sexual health education curriculum that covers sexually transmitted infections, contraception in general, and condom use. Michigan law does not require sex education be taught in schools; decisions about sex education are left up to individual school districts.

Some of the participants also indicated that young women are not always valued and appreciated compared to men or women in powerful positions, especially in terms of support that is practically demonstrated. Their concerns whether related to their healthcare needs or other social services may not be taken seriously or followed up thoroughly. Several participants suggested offering venues and programs to promote creativity and give girls a voice.

*Having help from schools, from healthcare, from the state, from just having overall support I think to be taken seriously, because I think a lot of women—and I mean it seems like with what I’ve seen especially in Flint, there aren’t a lot of kids that are involved in different things, but I think it’s hard to understand that your voice does matter, cuz I know that’s what I went through. It’s just like, “Oh, I’m talking too much. I don’t need to do stuff like that.”* **College student, 24**

Some of the young women interviewed indicated that having creative outlets for young girls would also reduce their vulnerability to engaging in risky activities out of boredom.

Participants working with trafficked women reported that there is a high prevalence of human trafficking in Genesee County. They said that traffickers often target girls coming from unstable households or households with financial problems, or girls who are runaways or who are justice-involved. Limited efforts to protect young and vulnerable women increases risk. Respondents indicated that early identification and intervention with at-risk girls is critical.

*It was a 19-year-old, she had a pimp who was following her, and beating her up, and controlling her. She ran, and he tracked her down, pulled a knife on him, and he took the knife, stabbed her, killed her, stabbed like 20 times, 26 times, and then slit her throat... I didn’t really think about that case as really anything other than a murder case until I started to get some education about human trafficking. Then kind of the idea was what if—we knew what happened to her at 19, but what happened to her at 15*? **Male attorney, 52**

### Perinatal women and new mothers

Suggestions to better meet the needs of perinatal women and new mothers in the Flint area included better access to healthy food and to transportation, streamlining access to social services and other resources, childbirth and new baby information and support, better employment and childcare options, and dignity in reproductive healthcare.

#### Healthy food and transportation

Access to basic needs such as clean water and healthy food was underscored as important unmet needs for this group. Access to healthy food was a challenge since most of the existing grocery stores are located outside of the City of Flint. Transportation to grocery stores were said to be difficult as perinatal women and mothers may face difficulty navigating the public transportation system with young children and groceries, especially during winter seasons.

*We have poor access to quality foods here because of the geography of the place as well as income. Then the bus system is pretty inconvenient. For most of the routes you have to travel all the way downtown and then back up. I just can’t imagine going to a grocery store, purchasing bulky fruits and vegetables with one or two kids in tow, and a baby.* **Doctor, 55**

Poor access to transportation also limits access to other services including those freely available to mothers in the community.

*… we could teach parenting classes all day long and set up shop in numerous places, but when you have a new baby and no transportation, you’re not gonna carry that baby to Downtown Flint*. **Male social worker, 33**

Transportation challenges were also mentioned as a barrier to attending prenatal and postnatal appointments, especially for new mothers.

#### Streamlining access to social services and other resources

Some mothers said that the steps followed for obtaining social services are lengthy and often do not take into consideration that women may need to utilize these services frequently. The difficulty of navigating social service systems can discourage women from accessing the services they need and qualify for.

*Let’s say she needs diapers. I sit down with them and we find a community resource for it. You have to prove that you need it. You have to get the bus route, find a bus pass, get on the bus, go down there, show your social security card, show your Medicaid card, sign this, sign that, get a pack of diapers and then go. Then you use those diapers, then you have to come back, but you can’t use it too much. Even though you still got the same job, but miraculously something has to change by next month or if you use it too much, then we’re going to say you’re a drain on the system. There are so many steps just to get basic, simple help that it just beats it down. I’m an educated mom. I have special needs kids. I adopted out of foster care. I have filed 15 appeals and grievances just to get my kids basic support. I have spent thousands and thousands of dollars hiring advocates for special education.* **Social worker, 43**

#### Childbirth and new baby information and support

Participants shared that in many parts of the community, motherhood is portrayed as flawless. Therefore, new mothers are surrounded by unrealistic expectations regarding their role as a mother. As a result of this social pressure, perinatal women are reported to be at risk of developing anxiety and depression or difficulties bonding with their newborn. Participants suggested having mentor mothers who will give practical support and realistic information about motherhood. Participants also proposed teaching perinatal women about real pregnancy and childbirth experience and initiating parenting education during pregnancy. They said such interventions may help correct unrealistic expectations around pregnancy, childbirth and motherhood. To this end, providing nonjudgmental care for young mothers and home-based postnatal follow-ups were recommended. Some of the participants mentioned “Moms Bloom” program, a volunteer run program in another county where new mothers receive domestic help and childcare by mothers from the community. Some of the participants said that the emphasis on contraceptives given by health care providers for women in the postpartum period is inadequate, leading to repeated pregnancies.

#### Employment and childcare

Participants suggested that women have limited job opportunities in the perinatal period, contributing to poverty and inability to access other services. They said some employers do not consider pregnant women for jobs because they will eventually take time off. To address this, participants recommended employment support that does not discriminate against pregnant women or a system that does not consider pregnant women as a liability.

Almost all of the participants explained that new mothers struggle with lack of affordable, accessible and quality childcare. All of the participants agreed that childcare facilities available are expensive and numerically very few. This is further complicated by poor access to transportation and lack of paid maternity leave in most jobs. Many women in the area work on short-term contracts, which often do not provide paid leave, healthcare and other benefits. Participants recommended that employers should provide a minimum of 3–6 months paid maternity leave.

The need for ***dignity in reproductive healthcare*** during the perinatal period was raised by some participants. Participants stated healthcare practitioners sometimes provide unsolicited advice to new mothers about their reproductive health choices such as the number of children they should have. Such comments may discourage or delay engagement with health care. The need for a system that respects the choices of women in this period was highlighted.

Respondents also explained that many healthcare facilities are not set up for perinatal women visiting with young children, potentially leading to limited access to healthcare. Thus, the need for safe and accommodative healthcare waiting rooms was emphasized. Participants added that home-based postpartum checkups and follow-ups may contribute to better health of new mothers and newborns.

Some of the women also shared their dissatisfaction with the care they received during their own pregnancy and birth experiences.

*It became really apparent to me after I had [baby name]. I had him at [name of hospital]. As soon as he was out of me they didn’t care about or for me anymore. I was glad that he was being prioritized, but I just had an emergency C section. To be left alone in a room for an hour afterwards is unacceptable. I think women need to be shown that they’re genuinely cared about when they’re pregnant and that it isn’t about the unborn baby, it’s about their value as a person and probably in general women need to understand more and be more valued as people*. **Volunteer working with postpartum women, 28**

Finally, participants working with pregnant women described stigma towards pregnant women using substances. They shared that practitioners including the nurses, lab technicians and the doctors are compelled to give advice or look down on pregnant women using substances, which discourages them from seeking the needed treatment. One of the participants shared a quote from her client as *“Nobody loves a pregnant junkie.”* Participants underscored the importance of a healthcare system that treats all women with respect and is sensitive to the needs of these vulnerable women with substance use issues, in order to better engage them in care. Moreover, from the healthcare providers’ side, having a one-stop shop that specializes in pregnancy and mental health was also recommended.

### Older women

Identified needs of older women included access to healthy food, transportation, healthcare, continued employment opportunities, friendship, and opportunities to contribute.

#### Healthy food, transportation, and healthcare

Almost all of the participants agreed that similar to perinatal women, older women also struggle from poor access to healthier meals and experience transportation challenges to attend their doctor appointments.

Participants also noted that older women need good and reliable access to healthcare. However, many older women suffer from lack of quality and affordable healthcare services. Some of the older women interviewed also said that they had to postpone retirement or get other employment due to high healthcare related expenses. Often, the choices they have to make are between getting their pain prescriptions, healthy food, or paying their rent and staying housed. Participants recommended that elders needed to be able to have lower prescription costs or free medical care, free prescription coverage, to help offset that.

Older women were also reported to have poor access to services available in the community including healthcare. Majority of participants mentioned that older women on Medicare often report that their insurance does not cover well or the eligibility requirements are very stringent.

*There’s so much that Medicare, and Medicaid require of these people that they can’t do, and there’s so much red tape, and policy that you have to follow that is unrealistic. Somebody needs help. Somebody needs healthcare. If somebody has a stroke, and they can’t walk, you would think that their insurance would pay for assistance, and they don’t*. **Social worker, 32**

#### Employment opportunities

Participants said that job opportunities available to older women are limited and often subtle discrimination on the basis of age was said to be common. Participants also said that employment opportunities for older women decline with age. As a result, older women who do not have other forms of social support face challenges to access the available state assistance.

*They [older women] have to try to receive assistance from welfare – from Social Services, and they need support. They really need support. A lot of ‘em are not able to – when they get in that 50-ish range, they’re not always hired. Even though they may have a skill, they’re not hired because of their age, so they need support in those areas, especially if they don’t have family support. Sometimes, there are women who don’t have siblings or children, and they’re out there alone, and they just need help*. **Community member, 72**

Some of the older women emphasized the importance of ***affordable, quality housing***. Many older women are housed in older houses which often require frequent maintenance which may not be affordable for many older women. Therefore, participants underlined the need for readily available and affordable home repairs for older women.

#### Social support, friendship, and opportunities to contribute

Respondents suggested that many older women in the community are lonely, have poor social support, and struggle to maintain lasting relationships due to people leaving the area and the death of peers. They reported that many longstanding neighborhoods are being disrupted due to outmigration. As a result, many older women do not have adequate social support to tap into in times of need. To address the problem of loneliness, respondents suggested home visits focused on homebound older women and introducing elderly-focused community activities. Understanding older women’s desire to contribute and creating avenues for their contributions (i.e. matching older women with young girls) was suggested to increase their sense of purpose and enable them build their legacy. Many participants highlighted the importance of intergenerational interaction between older and younger women. Such interactions may give older women a voice, hope and restore their faith in themselves:

*The older women in Flint– they need so that their voice be heard. They need a voice. A lotta the older women are told your information is outdated, so they just kinda go away. That gives you a reason to thrive, to live. If you're 70, and you know that Susie and Martha and Tasha are coming to sit at your feet and hear your stories, that gives you a reason to keep going. They need to let be known that they value – they are valued – excuse me – in this community.* **Social worker, 30**

Some of the participants said that older women have a lot to offer but they need a platform where they can share their skills or offer their service to others.

…*they [older women] give a lot, have given a lot to the community. They have a lot of wisdom and knowledge on what to do, how to do, and a lot of that has just been put to sleep and nobody wants to know. Nobody really cares and it’s just been put to sleep and just go ahead on and say, “Well, I could,” or “I know how to do that,” or “I could do that, but nobody ever asked me, you know. So, I just go to sleep with it,” you know*. **Elder, 80**

Participants suggested that being engaged, remembered, and needed improves mood and meaning, and that lacking these things can lead to depression.

*I primarily think of the mental health needs like increased depression, decreased life span, increased health issues. If not depression, then perhaps lack of meaning in life. Maybe not depressed but just feeling like, “I’ve had a profession, or I’ve raised my kids, and what good am I doing in the world now?” Just not having the same level of meaning in their lives that they might have had during younger years. Often people think about when an elderly person needs something, “I need to go shovel their driveway or maybe take a meal.” It’s not so much, “I wanna be their friend,” because the elderly population in our culture are just, in many ways, ignored and marginalized.* **Social worker, 49**

Increased awareness of the resources available (healthcare, jobs) in the community was suggested to address the needs of older women. Moreover, reaching out to other women and support each other and having a network of other women supporting them was proposed.

### Women with disabilities

This category involved women with hearing impairment, visual impairment, intellectual and physical disabilities. Identified needs included improved transportation, improved physical infrastructure of the city, more available care and treatment services, and authentic inclusion.

#### Transportation, physical infrastructure and accessibility

Transportation difficulties were mentioned as a shared challenge among women with disabilities of any group. Women who use wheelchairs experience exceptional difficulties during the winter.

*I’ve seen this woman, she didn’t have any legs. She was in a wheelchair. This was back when it was snowin’ really, really bad. Her wheelchair got stuck and everybody was just goin’ down the street. When I see she was in a wheelchair, she had a power wheelchair. … I seen her so I went, and she’s like, “How did you know I was stuck?” I said, “Cuz you wasn’t movin’ anywhere. It’s snowin’ really hard, so I just came and I moved her. I’m just sayin’, I’m pretty sure she usually gets around however she gets around.* **Patient navigator, 40**

Flint’s declining infrastructure was mentioned to be the main challenge to mobility for women with disabilities. Many of places around the city do not have sidewalks. Some of the streets do not get plowed or treated regularly during winter. Classrooms in some of the colleges in the city are not wheelchair accessible. Thus, participants mentioned a need for renovation of existing physical infrastructure to increase accessibility for women with physical challenges, and the need for additional transportation services that would pick women up, especially during the winter.

Participants also suggested that having service animals could help for security of women with disabilities in the city. In addition to service animals, access to affordable technologies/devices such as electronic doors (for women with mobility challenges, visual impairment) and doorbells that have flashing lights (for women with hearing impairment) were reported to be a gap.

*I dealt with the deaf, so I know a lot of deaf people. They'd want the doorbell that flashed because they would not know anybody was at their door. That was security for them, knowing someone's standing at their door and then they could look through—as long as they could see, they could look through the hole, peephole, and know who's out there or what's out there. The phone flashing, just having the available devices to keep, make them feel safe in their home and environment*. **Community member, 63**

#### More available care and treatment services

Caregivers expressed a need for more daycare for girls and women with disabilities. They said that summer programs are helpful, but are often expensive and only short-term. Some respondents also mentioned that accessing services for their children with disabilities is a challenge because of co-occurring conditions:

*I have another, my younger daughter is—she has both a developmental disability and a mental illness. She can’t get a lot of services geared towards mental illness because she’s developmentally delayed. They say, because you’re developmentally delayed, I don’t know, you can’t have mental health services. Then, there’s a lot of developmentally delayed services that she can’t get because she’s too mentally ill. She’s caught in the middle because it just, you’re not supposed to have both. However, foster care produces both. In the same person.* **Volunteer, 28**

#### Authentic inclusion

Participants mentioned the importance of authentic inclusion of women with disabilities in all aspects of the community including the media. Moreover, creating platforms for women with disabilities to connect with others and share their stories and providing help that considers their dignity and pride was recommended.

### LGBTQIA women

Needs mentioned for LGBTQIA women included reduced discrimination, especially in housing, and improved provider training and sensitivity to their needs.

#### Reduced discrimination

Participants described LGBTQIA women as “*the most hated and the most misunderstood*” of all the groups of women in Flint. They remarked that there is especially high stigma, racism and sexism toward trans-women of color. They also reported that LGBTQIA women face isolation and exclusion at work places because straight men do not feel comfortable around them.

*“They’ve been too long ignored and lost and neglected. Their needs are huge. They need to be heard and seen”.* **Librarian, 50**

Introducing “meet and greet” programs where LGBTQIA women can build support networks and help break stigma. Early intervention, such as interventions at school levels by diversifying sex education to include LGBTQIA was suggested to reduce stigma.

#### Housing

Respondents explained that LGBTQIA women in the area often face difficulties securing housing (including renting a house) due to discrimination in the rental market and sometimes even in shelters. Participants indicated that LGBTQIA women are unwelcome in many religious or faith-based homeless shelters.

*… housing the LGBTQ youth, very, very few resources. …There’s just not a lot of acceptance. Even staying in a communal living environment, they have a lot of hurdles to overcome. I’ve even had a staff that—we have sensitivity training and stuff. In the past, “Well, this goes against my religious view.” Well, I’m not asking you. I don’t employ you for your religious view. This person is homeless. I don’t care. I’ve had clients sleeping in the parking lot of Meijer’s in the dead of winter in their car that doesn’t start. Who cares? They’re homeless. That’s what we do. They have a lot of hurdles. There’s very little, in my opinion, compassion for that whole entire population in our community.* **Leader of a homeless shelter, 40**

Therefore, they face exceptional housing challenges since the services available to them are extremely limited. LGBTQIA women also face difficulties during discharge from other institutions psychiatric facilities, correctional facilities and substance use recovery programs as the number of homeless shelters that are willing to admit them are fewer.

#### Better provider sensitivity and training

Participants stated that the some of the existing health and welfare service providers are not trained to work effectively with and be sensitive toward the specific needs of LGBTQIA women. Moreover, the services available to this groups are described as “woefully under-resourced”. Therefore, participants described the group as “the most under-served”.

*It’d be nice if they opened up a clinic for that community. Because often, they don’t want to go to—okay, if you’re female who does not identify as a female, do you go to an OB/GYN? You don’t know. I don’t know if you would. I don’t know if you wouldn’t. That would be nice if their need was met in the medical field, to not be so intimidating of like, “I don’t want to go to the doctor.”* **Graduate student, 24**

Lack of understanding and sensitivity from the providers,

*This is an area, just a general gap in services that we’ve seen. Service providers are not educated on providing services to the LGBTQ…* **Homeless shelter staff person, 38**

Due to these gaps in the healthcare system, participants noted that LGBTQIA women may access inadequate care or their needs may remain unaddressed. Thus, participants suggested that having specialized clinics with sensitive providers might improve LGBTQIA women’s access to healthcare. Providing health and human service providers with a menu of services appropriate for LGBTQIA women and educating them on how best to serve this population was proposed to improve their access to services and health outcomes.

## Discussion

This qualitative study utilized intersectionality theory as a framework to understand barriers and facilitators of improving wellbeing and increasing access to services among Flint women. We focused on needs of women who are young, perinatal, new mothers, older, disabled, and LGBTQIA women. The results reflected the multiple challenges experienced by women in different life-stages, disability status and gender identities and sexual orientation. These disadvantages posed a significant challenge even to meeting the most basic needs. Several overarching themes affecting overall health and access to care, including (1) limited social support, (2) difficulties with mental health and feelings of self-worth, (3) inadequate resources for women’s health and (4) significant structural barriers within the built environment were identified.

### Need for social support

One of the most widely cited needs across all of the populations surveyed was the need for increased connection between individuals. Several respondents discussed the need for mentorship as it relates to young women and older women, while the need for inclusion was highlighted for LGBTQIA and women with disabilities. Research indicates that social discrimination is a primary driver of disparities in mental health outcomes in individuals from marginalized groups [23, 24]. People from special populations experience more stigma which, in turn, creates greater disruption in social networks and connectedness, impeding the development of successful interpersonal relationships [23]. Conversely, receiving greater support may be specifically helpful for individuals with multiple disadvantages [25]. For instance, research suggests that elderly African American women with greater social interconnectedness lived longer than social-isolated women, controlling for health and socio-economic status [26].

The need to create these social structures in order to promote the physical health and wellbeing of vulnerable women is apparent. Fostering interpersonal support through formal mentorship programs was suggested to link individuals across special populations (e.g. perinatal women and older women, young women and older women). These results support existing programs such as *Smart-Girl* [27], which matches adolescents of color from low-resource communities with “near peers,” “guides,” and “coaches” (older girls and women from similar backgrounds) to discuss issues commonly faced by individuals from vulnerable groups, such as bullying, body image difficulties, and stigma. Younger participants benefit from interacting with developmentally-advanced women who can draw on similar lived-experiences, while mentors strengthen their own skillset in addressing stressful life events. More informal connections between individuals can also promote increased well-being. Research suggests that women with informal female mentors report greater friendships and social interactions compared to women in more formal mentoring relationships; moreover, women protégés reported greater social support from female mentors relative to male mentors [28].

### Need for increasing sense of self-worth and improved mental health

Perhaps not surprisingly, our findings also suggest women from stigmatized groups experience greater mental health needs and have lower feelings of self-worth and appreciation. It was noted that younger women needed to be “loved” and “appreciated” while older women need to “increase their sense of purpose.” The intersectionality framework suggests that individuals who identify as coming from multiple special populations (e.g. racial/ethnic sexual minorities or perinatal women from low-resource communities) experience greater levels of stress relative to individuals from a single special population. In turn, this increased exposure to stress leads to greater psychological distress [23] and subsequent increases in mental health problems. Our findings from interviews with young women confirm that lower self-esteem and self-acceptance may lead to engaging in riskier behaviors with longer-term negative implications (i.e. substance use, unprotected sexual experiences, etc.), creating a negative spiral of mental health problems.

In addition to greater access to mental health services, our results suggest that self-worth and mental health may be improved by increasing the number of positive activities available to women in special populations. Specifically, interviewees suggested creating more creative outlets to decrease boredom and provide prosocial alternatives to engaging in riskier behaviors. While disadvantaged communities are less likely to have or maintain expensive recreation facilities [29], a robust literature suggests even low-cost activities requiring limited resources (such as walking) are associated with increases in well-being [30]. Our findings support modest investments in infrastructure (such as community centers, parks, and sidewalks) to foster opportunities for positive activity within the community as a way to address the needs of vulnerable women.

### Need for improved healthcare resource

Another central theme shared by all of the populations was the negative impact of disparities in access to care, especially the lack of services that address the unique needs of individuals from special populations. Participants pointed to limited access to health-related information, including comprehensive information on contraception options and sexual health, as a problem for young, perinatal and LGBTQIA women. Participants also identified a lack of dignity in healthcare across marginalized populations (specifically for LGBTQIA, and both perinatal and new mothers) as a proximal risk factor for not engaging in necessary services. These results replicate related research that suggests marginalized individuals face greater health care stigma which leads to the underutilization of health care services, including foregoing regular check-ups and necessary testing [31, 32]. These lapses in care, in turn, have real effects on both illness progression and overall well-being [33]. Increasing home-based care, subsidies for medical services and medications, increasing the number of specialized clinics that serve vulnerable populations were suggested. These suggestions are supported by previous research that stated special populations report higher levels of satisfaction with specialized care [34]. Therefore, adapting a flexible approach to healthcare services that can accommodate the needs of individuals from special populations might be relevant.

### Need to decrease structural barriers to wellbeing and accessing care

Participants reported a number of existing structural barriers that interfere with general wellbeing and accessing care across all groups. Specifically, women noted the continued need for improved access to clean water and healthy foods, a well-documented problem in the Flint community [35, 36]. Participants also indicated that inadequate transportation is likely to prevent new mothers and older women from accessing services that were available in their community, such as doctors’ offices and social welfare agencies. Women with disabilities may face additional challenges to utilizing accessible transportation in the winter, when streets and sidewalks may not be plowed due to limited financial resources city-wide. Moreover, lack of accessible and affordable housing was also raised. Respondents reported that Stigma may compound housing shortages, especially for LGBTQIA women who may be prevented from accessing services like homeless shelters because of discrimination. These findings are consistent with the extant literature that point to the central role of barriers within the built environment to accessing care [37] and how these barriers may disproportionately impact women from special populations [38].

Participants pointed to the need to increase community-level supports to help women navigate around these barriers. One way to reduce these disparities could be the use of community-based navigators who help guide women through these steps [39, 40]. Many respondents also identified a need for further low-cost programs to address the specific structural needs of the community, such as increasing accessibility and affordability of housing and home repair services or improving snow removal services.

Our results are also consistent with the previous studies that reported the issue of human trafficking in the state of Michigan. Human trafficking is a growing concern in the state of Michigan [41]. The state is particularly at risk of human trafficking because its shared international border, industrial agriculture, history of adult prostitution zones and a huge presence of immigrant population [42].

### Strengths, limitations, and future directions

This study has several strengths, including drawing qualitative data from a relatively large sample of individuals from a traditionally underrepresented area and examining a variety of special populations. Moreover, the study utilized Community-based participatory research (CBPR) principles and academic-community partnerships to include community members in the collection, analysis and reporting of findings. These results, however, should be considered in light of several limitations. We included the perspectives of all women participating in the study in addressing the needs of vulnerable groups. Although it was relevant for the focus of this study, we did not specify the identities of participants across the lines of ethnicity, disability, race and other identifiers. This was because Flint is a small community and further qualifying the information would possibly compromise identities of the study participants.

Future research may want to limit responses to only the specific voices of women in these marginalized groups. Moreover, we collected responses at only a single time point. Flint is a dynamic community that is influenced by the national attention received following the Flint Water Crisis; future research should consider the ways these perspectives change as the city grows.

## Conclusion

Efforts directed towards improving women’s health and wellbeing should include perspectives and suggestions of diverse groups of women from the community. Acting on suggestions that emanate from the community’s lived experiences may reduce inequalities in health and wellbeing.

## Data Availability

The data is available from the corresponding author on reasonable request.

## Abbreviations

CERB: Community Ethics Review Board
LGBTQIA: Lesbian, gay, bisexual, transgender, queer, intersex and asexual
